# Functional PIN1 promoter polymorphisms associated with risk of nasopharyngeal carcinoma in Southern Chinese populations

**DOI:** 10.1038/s41598-017-04156-z

**Published:** 2017-07-04

**Authors:** Liuyan Zeng, Shengqun Luo, Xin Li, Mengxuan Lu, Huahui Li, Tong Li, Guanhua Wang, Xiaoming Lyu, Wenrui Jia, Zigang Dong, Qiang Jiang, Zhihua Shen, Guo-Liang Huang, Zhiwei He

**Affiliations:** 1 Department of health management center, the affiliated hospital of Guangdong Medical University, Zhanjiang, Guangdong, China; 2 China-American Cancer Research Institute, Dongguan Scientific Research Center, Guangdong Medical University, Dongguan, China; Key Laboratory for Epigenetics of Dongguan City; Key Laboratory for Medical Molecular Diagnostics of Guangdong Province, Dongguan, China; 3 Cancer Research Institute, Southern Medical University, Guangzhou, 510515 China; 4 Department of laboratory medicine, the third affiliated hospital of Southern Medical University, Guangzhou, China; 5 School of Laboratory Medicine, Guangdong Medical University, Dongguan, China; 6 The Hormel Institute, University of Minnesota, 801, 16th AVE, NE, Austin, MN 55912 USA

## Abstract

Our previous work reported the association between two single nucleotide polymorphisms (SNPs) in PIN1 promoter and nasopharyngeal carcinoma (NPC) risk with a small sample size in a low incidence area. This study investigated the association between the two SNPs and NPC risk in 733 patients and 895 controls from a high incidence area. The results indicated the genotype and allele frequencies of -842G > C and -667C > T were both significantly different between patients and controls even using the resampling statistics. The -842GC and -667TT genotypes showed a significantly increased risk of NPC (OR = 1.977, 95% CI = 1.339–2.919, *P* = 0.001 and OR = 1.438, 95% CI = 1.061–1.922, *P* = 0.019, respectively). Compared to the most common -842G-667C haplotype, -842G-667T haplotype and -842C-667C haplotype showed a significantly increased risk of NPC (OR = 1.215, 95% CI = 1.053–1.402, *P* = 0.008 and OR = 2.268, 95% CI = 1.530–3.362, *P* = 0.001, respectively). Further reporter gene expression suggested that variant -842C-667C and -842G-667T were associated with an enhanced transcriptional activity. In conclusion, our findings suggest that -842G > C and -667C > T in PIN1 promoter are associated with NPC risk; as well as the promoter activity is mediated by functional PIN1 variants.

## Introduction

Peptidyl-prolyl cis/trans isomerase (*PIN1*) is a highly conserved enzyme that binds to and isomerizes specific phosphorylated serine or threonine residues preceding proline (Ser/Thr-Pro). PIN1 has been reported to be overexpressed in several types of human cancers^1^. Its induction of conformational changes of Pro-directed phosphoproteins potentiates multiple oncogenic signaling pathways^2, 3^. Through the regulation of diverse oncogenic signaling pathways including growth-signal responses, cell-cycle progression, cellular stress responses, neuronal function and immune responses, PIN1 plays an important role during cancer development^4^. For instance, PIN1 contributes to the development of aggressive prostate cancer by abrogating the AR-catenin interaction and thereby increasing beta-catenin coactivation of Tcf4^5^. PIN1 deletion suppresses the induction of cyclin D1 by oncogenic activated Neu or Ras in mammary tumorigenesis^6^. Moreover, PIN1 has been reported to be involved in the regulation of several viruses, such as human immunodeficiency virus type I^7, 8^, Kaposi’s sarcoma-associated herpes virus^9^, hepatitis C virus^10^, and Epstein-Barr virus^11^. By interaction with EBV DNA polymerase BALF5 subunit, PIN1 might modulate EBV DNA polymerase conformation for efficient viral DNA replication^11^.

Nasopharyngeal carcinoma (NPC) is an Epstein-Barr virus (EBV)-associated epithelial malignancy^12^, with an unusual disparity in ethnic and geographical distributions^13^. Although it is an uncommon disease in most countries, the prevalence rate of NPC is remarkably high in Southern China, especially in Guangdong Province^14, 15^. Over 95% of NPC is associated with EBV. The severity of EBV infection is related with carcinoma type: undifferentiated carcinomas have the highest EBV titers^16^. For the important role of PIN1 in EBV and multiple oncogenic signals, it is reasonable to postulate that PIN1 plays a key role in NPC. Recent study demonstrates that overexpression of PIN1 enhances cancer growth and aggressiveness in EBV-associated NPC^17^.

Our previous work indicated that two PIN1 polymorphisms were associated with the risk of NPC in a relative small sample from Hunan province, not a typical high incidence area of NPC^18^. Therefore, studies with larger population in high incidence region of NPC are needed to verify previous observation. In this study, associations between the two SNPs and the risk of NPC were evaluated in 733 patients and 895 controls from Guangdong Province, and the promoter activity that may be mediated by potentially functional PIN1 variants was assessed in NPC cells.

## Results

### Characteristics of the study population

A total of 733 nasopharyngeal cases and 895 controls from Guangdong province in Southern China were recruited in this study. Main characteristics of the study subjects were presented in Table 1. There were no statistically significant differences in the distributions of age (*P* = 0.052) and gender (*P* = 0.131) between cases and controls.

**Table 1:** Characteristics of nasopharyngeal carcinoma patients and controls.

Characteristics	Patients	Controls	*P* value
Age			
<45	309	335	0.052
≥45	424	560	
Gender			
Male	440	504	0.131
Female	293	391	
Primary tumor extension			
T1 + T2	89	—	
T3 + T4	146	—	
Lymph node status			
N0	28	—	
N1 + N2 + N3	207	—	
Metastasis			
NO	211	—	
YES	26	—	

### Distribution of PIN1 polymorphisms and risk of nasopharyngeal carcinoma

The genotype and allele frequency distributions of PIN1 SNPs (-842G > C, rs2233678 and -667C > T, rs2233679) in patients and controls were showed in Table 2. The observed genotype frequencies for these two SNPs were in agreement with Hardy-Weinberg equilibrium in the control subjects (*P* = 0.095 for -842G > C and *P* = 0.058 for -667C > T, respectively).

**Table 2:** Genotype and allele distribution of -842G > C and -667C > T in patients and controls.

Polymorphism	Patient	Control	*P* value	OR (95% CI)
-842G > C				
Genotype				
GG	660 (90.1)	848 (94.8)	0.001	
GC	69 (9.4)	45 (5.0)		
CC	4 (0.5)	2 (0.2)		
GC versus GG			0.001	1.977^a^ (1.339–2.919)
CC versus GG			0.287	2.520^a^ (0.459–13.823)
CC + GC versus GG			0.001	2.000^a^ (1.366–2.927)
Allele				
C	77 (5.3)	49 (2.7)	0.001	1.970 (1.367–2.838)
G	1389 (94.7)	1741 (97.3)		
-667C > T				
Genotype				
CC	241 (32.9)	319 (35.6)	0.048	
CT	359 (50.0)	453 (50.6)		
TT	133 (18.1)	123 (13.8)		
TT versus CC			0.019	1.438^a^ (1.061–1.922)
CT versus CC			0.667	1.049^a^ (0.844–1.303)
CT + TT versus CC			0.258	1.127^a^ (0.916–1.385)
Allele				
T	625 (42.6)	699 (39.1)	0.038	1.160 (1.008–1.335)
C	841 (57.4)	1091 (60.9)		

^a^Data were calculated by unconditional logistic regression with adjustment for age and gender.

The genotype frequencies of PIN1–842G > C and -667C > T were both significantly different between cases and controls (*P* = 0.001 and *P* = 0.048, respectively, Table 2). Compared to the -842GG genotype, the -842GC heterozygote was associated with a significantly increased NPC risk (OR = 1.977, 95% CI = 1.339–2.919, *P* = 0.001, Table 2), whereas the -842CC homozygote with a statistically insignificant NPC risk (OR = 2.520, 95% CI = 0.459–13.823, *P* = 0.287, Table 2); combination of -842CC and -842GC genotype with a significantly increased NPC risk (OR = 2.000, 95% CI = 1.366–2.927, *P* = 0.001, Table 2). In the -667C > T SNP, the -667TT genotype offered an increased risk of NPC compared with -667CC genotype (OR = 1.438, 95% CI = 1.061–1.922, *P* = 0.019, Table 2). Interestingly, significant differences were shown in the distribution of both -842G > C and -667C > T allele frequencies between cases and controls. The -842C allele and the -667T allele were associated with the increased risk of NPC (OR = 1.970, 95% CI = 1.367–2.838, *P* = 0.001 for -842C allele and OR = 1.160, 95% CI = 1.008–1.335, *P* = 0.038 for -667T allele, respectively, Table 2). Further resampling statistics using monte carlo estimation indicated consistent results with the associations between the genotype and allele frequency distributions of the two SNPs and NPC risk (Supplementary Table S1).

The relationship between -842G > C and -667C > T genotypes and clinicopathological parameters were also analyzed with available data in some cases (Supplementary Table S2 and Supplementary Table S3). No associations were observed between genotypes in the two SNPs and clinicopathological parameters, including age, gender, primary tumor extension, lymph node status and metastasis in patients.

### PIN1 haplotypes and risk of nasopharyngeal carcinoma

**Figure 1:**
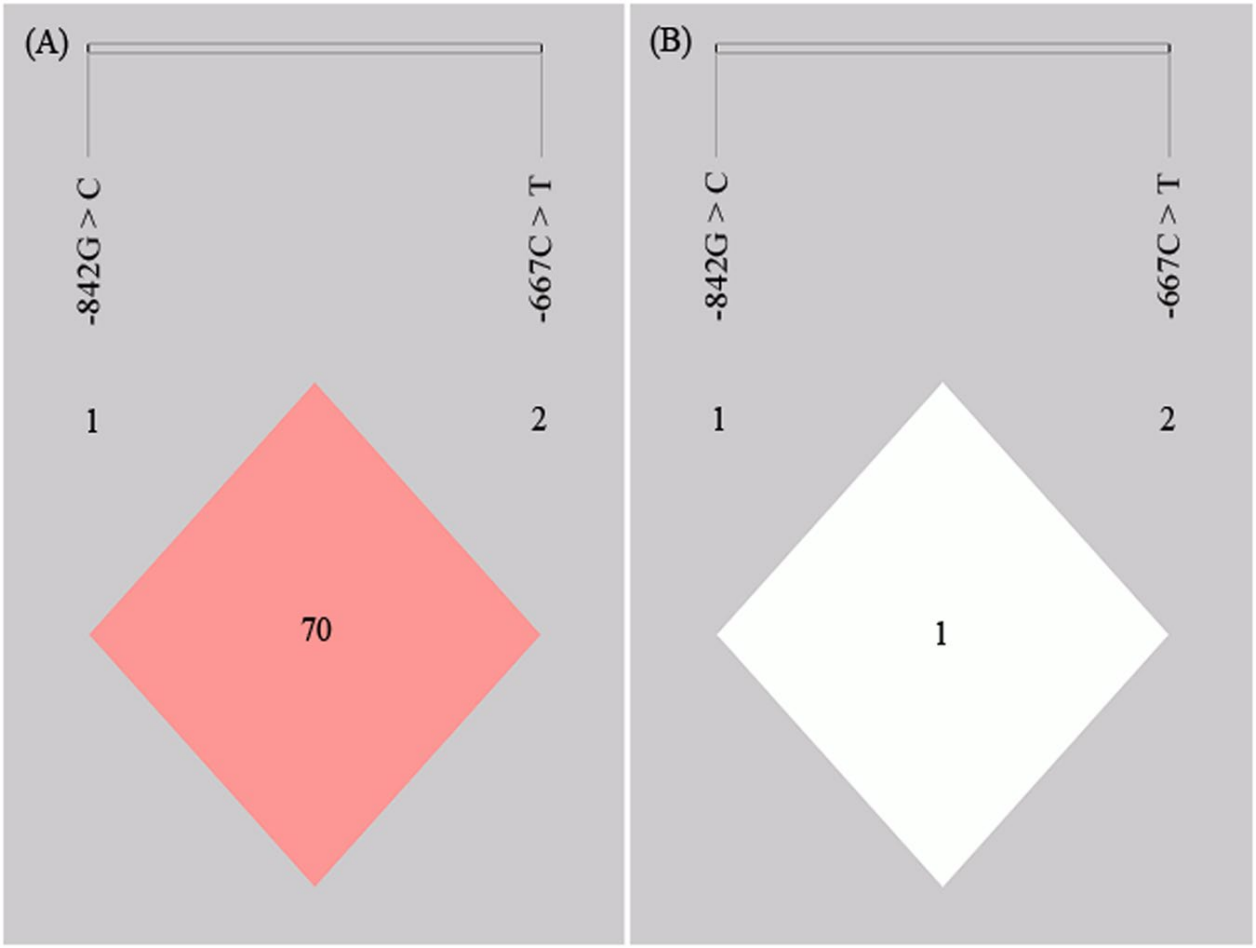
Linkage disequilibrium (LD) for the two SNPs of PIN1; (**A**) D′ and (**B**) r^2^ values.

The LD analysis showed that the two polymorphisms in the promoter region were not in LD (LD for PIN1 -842G > C and PIN1 -667C > T: *D*′ = 0.706 and *r* ^2^ = 0.014, Fig. 1). We evaluated the association between the risk of nasopharyngeal carcinoma and haplotypes constructed from the two PIN1 polymorphisms (-842G > C, -677C > T). Four possible haplotypes were obtained. The distribution of haplotypes was significantly different between cases and controls (P* < *0.001, Table 3). Compared to the most common -842G-667C haplotype, -842G-667T haplotype and -842C-667C haplotype showed a significantly increased risk of nasopharyngeal carcinoma (OR = 1.215, 95% CI = 1.053–1.402, *P* = 0.008 and OR = 2.268, 95% CI = 1.530–3.362, *P* = 0.001, respectively, Table 3); -842C-667T haplotype with a statistically insignificant NPC risk (OR = 1.361, 95% CI = 0.475–3.896, *P* = 0.565, Table 3). Since the sample size for -842C-667T haplotype was small, power calculation was performed for the specific statistical analysis. The result indicated a small power (8.9%) and the association analysis should be considered as inconclusive at this point. Larger sample size is needed to make a conclusion.

**Table 3:** PIN1 -842G > C and -667C > T haplotypes and nasopharyngeal carcinoma risk.

Haplotypes	Cases (1466 alleles) n(%)	Controls (1790 alleles) n(%)	OR (95%CI)	*P* value
-842G-667C	771 (52.6)	1049 (58.6)	1.00 (Ref.)	Ref.
-842G-667T	618 (42.1)	692 (38.7)	1.215 (1.053–1.402)	0.008
-842C-667C	70 (4.8)	42 (2.4)	2.268 (1.530–3.362)	0.001
-842C-667T	7 (0.5)	7 (0.4)	1.361 (0.475–3.896)	0.565
		P < 0.001^a^		

^a^Global test.

### Promoter activity of PIN1 polymorphisms

**Figure 2:**
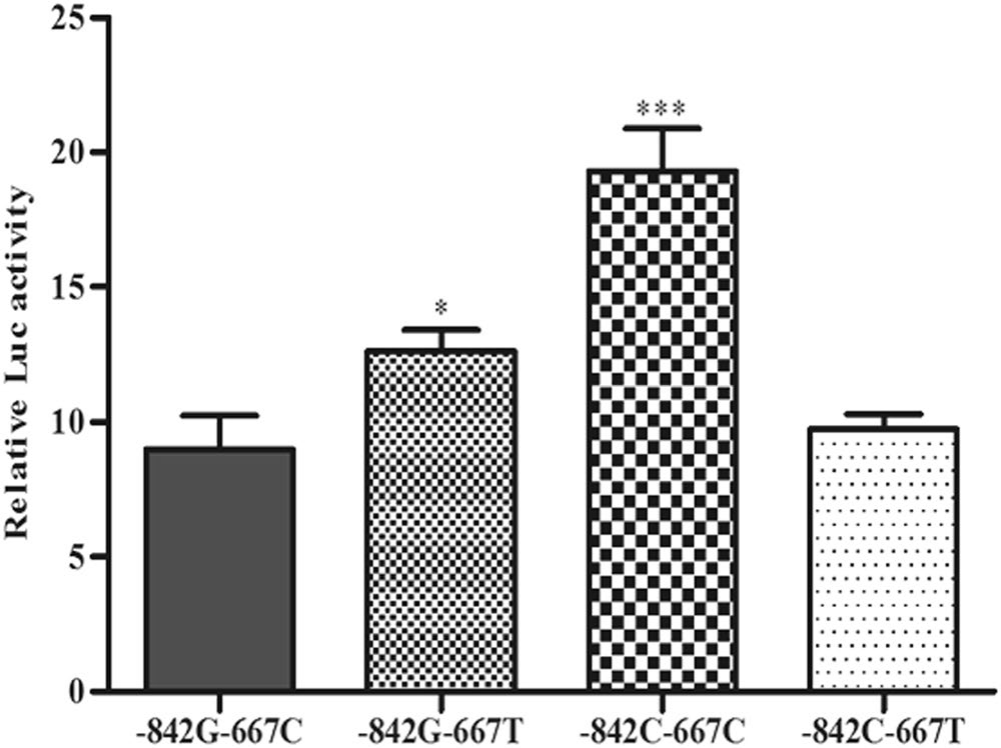
Luciferase activity of reporter gene expressions driven by the four haplotypes of PIN1. *P < 0.05, compared to -842G-667C; ***P < 0.001, compared to -842G-667C.

Four luciferase reporter gene constructs (-842G-667C, -842G-667T, -842C-667C and -842C-667T) were generated. The luciferase activity indicated that reporter gene expression driven by the variant -842C-667C and -842G-667T were both significantly higher than those driven by the wildtype -842G-667C (2.15 fold higher for -842C-667C, and 1.40 fold higher for -842G-667T, *P* < 0.05, Fig. 2). These results were consistent with the association analysis between the haplotypes and NPC risk. Although the reporter gene expression activity of the variant -842C-667T was slightly higher than that of the wildtype -842G-667C, the statistical difference was insignificant (*P* > 0.05, Fig. 2 **)**.

## Discussion

Inconsistent results were obtained from different studies on the association between *PIN1* polymorphisms and cancer risk. Several studies showed that compared to -842GG homozygote, -842GC heterozygote conferred a reduced risk of cancer, including breast cancer in non-Hispanic white women^19^, squamous cell carcinoma of the head and neck^20^, lung cancer in Southern and Eastern Chinese populations^21^ and NPC in Hunan Province of China^18^. However, from the data of a study on hepatocellular carcinoma, we inferred that -842GC heterozygote conferred an increased risk of cancer when compared to -842GG homozygote^20^. In this study, our finding showed that the -842GC and -842GC + CC offered a significantly increased risk of NPC in Guangdong Province populations. On the SNP -667C > T, most of the previous studies showed lack of statistical significance between genotype distribution and cancer risk. However, studies suggested that -667TT had a significantly increased risk of oral squamous cell carcinoma^22^ and hepatocellular carcinoma^20^. These data were consistent with our study, indicating that -667TT genotype was associated with increased risk of NPC. The discrepancy among studies could be attributed to the different types of cancers, heterogeneous ethnic background and various sample size. Our larger sample size from high risky area of NPC in Guangdong Province provided representative and stable assessment for the genotype and allele frequencies of PIN1 promoter.

A few studies have been reported to determine the association between cancer risk and PIN1 haplotypes. PIN1 haplotype -842C-667T was associated with decreased risk of squamous cell carcinoma of the head and neck compared to -842G-667T^23^. Haplotype -842C-667C was associated with reduced risk of esophageal carcinoma and lung cancer compared to -842G-667T^22, 24^. However, PIN1 haplotypes analysis in our study indicated that both -842G-667T and -842C-667C haplotype showed an increased risk of nasopharyngeal carcinoma compared to the -842G-667C haplotype.

Overexpression of PIN1 was found to enhance cancer growth and aggressiveness in NPC^17^. Functional analyses of the PIN1 promoter polymorphism with luciferase reporter assay were performed in our study. The result indicated that the transcription activity driven by the variant -842C-667C and -842G-667T were higher than those driven by the -842G-667C. The ratio of the transcription activity increasement of the two variant is consistent with the cancer risk ratio of corresponding haplotype.

Our data showed lack of statistical significance between -842G > C and -667C > T genotype and clinicopathological parameters including age, gender, primary tumor extension, lymph node status and metastasis in patients. The data was similar to our previous study^18^ and the study in breast cancer^25^. However, lack of statistical significance may be resulted from lack of clinicopathological information in some studies.

In conclusion, our study suggested that PIN1-842G > C (rs2233678) and -667C > T (rs2233679) polymorphisms were significantly associated with the risk of nasopharyngeal cancer in Guangdong Province, and the two SNPs might be a potential biomarker for cancer risk, especially for nasopharyngeal cancer. Diverse ethnic groups and further mechanistic studies of the different -842G > C and -667C > T variants are warranted to validate our findings.

## Materials and Methods

### Study population

The population based case-control study was conducted to assess the association of PIN1 polymorphisms with NPC susceptibility. The study population included *733* patients with nasopharyngeal carcinoma (NPC) and *895* healthy controls. Among them, a total of 336 patients with NPC and 895 controls were recruited at *Affiliated Hospital of Guangdong Medical University* (Zhanjiang, Guangdong Province, China) from September 2014 to October 2015. Another 397 patients with NPC were recruited at *Nanfang hospital, Southern Medical University* (Guangzhou, Guangdong Province, China) between July 2010 and December 2011. The patients were diagnosed by histopathology evidence and received no treatment before the blood drawing. The clinicopathological data including tumor size, nodal status and distant metastasis, clinically determined by computed tomography (CT) scan or magnetic resonance imaging (MRI), were available in only 235 patients (167 from Zhanjiang and 68 from Guangzhou). Controls were genetically unrelated cancer-free individuals matched with cases by age and gender. The mean ages of NPC patients and normal controls were 50.22 ± 15.91 and 47.05 ± 11.38, respectively. Informed consent was obtained from all participants. This study protocol was approved by the Ethics Committees of Guangdong Medical University.

### Genotyping analysis

Genomic DNA was extracted from peripheral blood samples on the basis of standard procedures using TIANamp Genomic DNA kit (Tiangen Biotech, Beijing, China). The two SNPs (-842G > C rs2233678, and -667C > T rs2233679) were genotyped using polymerase chain reaction-restriction fragment length polymorphism (PCR-RFLP) assay as described previously^26^. Since the two SNPs are close in distance, we used same primer sequences: sense 5′-CGG GCT CTG CAG ACT CTA TT-3′ and antisense 5′-AAA TTT GGC TCC TCC ATC CT-3′. The PCR products were digested with two different enzymes to identify the respective genotypes. Ban II (New England BioLabs, Beverly, MA, USA) was used for rs2233678 or Sac I (New England BioLabs, Beverly, MA, USA) was used for rs2233679, at 37 °C for more than 4 h or overnight. After that, the cleaved products were separated on 3% agarose gel and identified by ethidium bromide staining. The sequences of PCR products were confirmed by DNA sequencing in about 10% of the samples which were randomly selected.

### Construction of reporter plasmids

The pGL3 firefly luciferase reporter plasmids were constructed by Generay Biotech (Shanghai, China). The -842G/-667C reporter construct (wildtype promoter, namely -842G-667C) was prepared by amplifying the 1016 bp PIN1 promoter region (from −973 to +42 relative to the translation start site) and then ligating to the pGL3 basic vector (Promega, Madison, WI, USA). The -842C/-667C (namely -842C-667C), -842G/-667T (namely -842G-667T) and -842C/-667T (namely -842C-667T) reporter constructs were obtained from the -842G/-667C construct by site-directed mutagenesis. The reporter plasmids were confirmed by sequencing.

### Transient transfection and luciferase assays

The human nasopharyngeal carcinoma cells (*CNE2*) were cultured in RPMI Medium 1640 basic supplemented with 10% Fetal Bovine Serum, maintained in a humidified with 5% CO_2_ at 37 °C. Cells were seeded in 24-well tissue culture plates overnight for attachment before transfection and transfected using jetPEI™ Polymer-based DNA transfection reagent (Polypus-Transfection Inc, New York, USA) according to the manufacturer’s recommendations. Briefly, 1 μl of jetPEI™, 250 ng of firefly luciferase plasmid DNA (-842G-667C, -842G-667T, -842C-667C or -842C-667T), 2.5 ng of the pRL-TK vector (Renilla luciferase plasmid) was co-transfected as an internal control for transfection efficiency and diluted in 50 μl of NaCl (Polypus-Transfection Inc, New York, USA). Cells were incubated for 48 h after transfection. Luciferase activity was quantified by a Dual-Luciferase Reporter Assay System (Promega, Madison, WI, USA) using a FB12 luminometer (Titergek-Berthold, Pforzheim, Germany). Relative luciferase activity was calculated as a ratio of Firefly to Renilla signal intensity. The transfections were performed a minimum of three times, with samples in triplicate using different plasmid preparations for each transfection.

### Statistical analysis

The Hardy-Weinberg equilibrium was utilized to compare the observed frequencies with the expected genotype frequencies in the control groups. The different distributions of the genotype and allele frequencies of PIN1 were evaluated by chi-square test. The associations between the two SNPs genotypes and the risk of nasopharyngeal carcinoma were estimated by calculating the odds ratio (OR) and 95% confidence interval (CI), using the multivariate logistic regression analysis adjusted by age and gender. To validate the robustness of the conclusions, the genotype and allele distribution of -842G > C and -667C > T in patients and controls were re-analyzed by monte carlo estimation, a resampling statistics method, using the nonparametric test with P value and corresponding 95% confidence interval^27–29^. The chi-square test or Fisher’s exact test was performed in a subsequent analysis of the association between the genotype and clinicopathological characteristic in patients. We detected the two SNPs linkage disequilibrium (LD), analyzed haplotype frequencies between the two promoter variants and evaluated the association between the haplotype and nasopharyngeal carcinoma risk using SHEsis Online Version^30, 31^. Statistical analyses were performed with SPSS 16.0 software and *P* < 0.05 was considered to indicate statistical significance. Power analysis was performed in particular statistical test using PASS 11 software.

## Electronic supplementary material

Supplementary Information
