# Supplementary material for: Functional PIN1 promoter polymorphisms associated with risk of nasopharyngeal carcinoma in Southern Chinese populations
Source: Sci Rep. 2017 Jul 4;7:4593. doi: 10.1038/s41598-017-04156-z (PMC5496913; doi:10.1038/s41598-017-04156-z)
Supplement: Supplementary file 1 — Supplementary Information [file 41598_2017_4156_MOESM1_ESM.pdf]

Functional PIN1 promoter polymorphisms associated with risk of nasopharyngeal carcinoma in Southern Chinese populations

Liuyan Zeng [1, 2#], Shengqun Luo [2#], Xin Li [3], Mengxuan Lu [2], Huahui Li [2], Tong Li [2], Guanhua Wang [2], Xiaoming Lyu [4], Wenrui Jia [5], Zigang Dong [6], Qiang Jiang [3], Zhihua Shen [2], Guo-Liang Huang [2**], Zhiwei He [2*]

Authors' Affiliations: [1] Department of health management center, the affiliated hospital of Guangdong Medical University, Zhanjiang, Guangdong, China. [2] China-American Cancer Research Institute, Dongguan Scientific Research Center, Guangdong Medical University, Dongguan, China; Key Laboratory for Epigenetics of Dongguan City; Key Laboratory for Medical Molecular Diagnostics of Guangdong Province, Dongguan, China. [3] Cancer Research Institute, Southern Medical University, Guangzhou 510515, China. [4] Department of laboratory medicine, the third affiliated hospital of Southern Medical University, Guangzhou, China. [5] School of Laboratory Medicine, Guangdong Medical University, Dongguan, China. [6] The Hormel Institute, University of Minnesota, 801, 16th AVE, NE, Austin, MN 55912, USA.

[#] **These authors contributed equally to this study.**

[*] **Corresponding Author:** Zhiwei He, M.D., Ph.D., Professor, China-American Cancer Research Institute, Guangdong Medical University, No. 1 Xincheng Road, Dongguan 523808, China. E-mail: zhiweihe688@yahoo.com. Telephone: 86-0769-22896219.

[**] **Co-corresponding Author:** Guo-Liang Huang, Ph.D., Associate Professor, China-American Cancer Research Institute, Guangdong Medical University, No. 1 Xincheng Road, Dongguan 523808, China. E-mail: huanguoliang@gdmu.edu.cn. Telephone: 86-0769-22896405.

Supplementary Table S1. Resampling statistics of genotype and allele distribution of -842G>C and -667C>T in patients and controls

Polymorphism	Patient	Control	*P* value (95% CI for *P* value)
-842G>C			
Genotype			
GG	660 (90.1)	848 (94.8)	0.001 (0.000-0.002)
GC	69 (9.4)	45 (5.0)	
CC	4 (0.5)	2 (0.2)	
GC versus GG			0.001(0.000-0.002)
CC versus GG			0.404(0.380-0.429)
CC+GC versus GG			0.001(0.000-0.002)
Allele			
C	77 (5.3)	49 (2.7)	0.001 (0.000-0.002)
G	1389 (94.7)	1741 (97.3)	
-667C>T			
Genotype			
CC	241 (32.9)	319 (35.6)	0.047(0.037-0.058)
CT	359 (50.0)	453 (50.6)	
TT	133 (18.1)	123 (13.8)	
TT versus CC			0.021(0.011-0.031)
CT versus CC			0.702(0.678-0.726)
CT+TT versus CC			0.264(0.242-0.285)
Allele			
T	625 (42.6)	699 (39.1)	0.032 (0.026-0.039)
C	841 (57.4)	1091 (60.9)	

P value and corresponding 95%CI were calculated by monte carlo estimation.

Supplementary Table S2. Association between the genotype frequencies of -842G>C and clinicopathological characteristics of nasopharyngeal carcinoma patients

Parameters	-842G>C			*P* value
	GG	GC	CC	
Age				
<45	280	28	1	0.751*
≥45	41	380	3	
Gender				
Male	395	42	3	0.818*
Female	265	27	1	
Primary tumor extension[#]				
T1+T2	81	7	1	0.701*
T3+T4	129	16	1	
Lymph node status[#]				
N0	26	2	0	0.763*
N1+N2+N3	184	21	2	
Metastasis[#]				
NO	189	21	1	0.131*
YES	24	1	1	

* Two-sided Fisher's exact test was used.

[#] Data available only in some cases.

Supplementary Table S3. Association between the genotype frequencies of -667C>T and clinicopathological characteristics of nasopharyngeal carcinoma patients

Parameters	-667C>T			*P* value
	CC	CT	TT	
Age				
<45	93	160	56	0.347
≥45	148	199	77	
Gender				
Male	142	217	81	0.909
Female	99	142	52	
Primary tumor extension[#]				
T1+T2	27	44	18	0.242
T3+T4	56	71	19	
Lymph node status[#]				
N0	9	17	2	0.289*
N1+N2+N3	74	98	35	
Metastasis[#]				
NO	72	107	32	0.705*
YES	10	11	5	

* Two-sided Fisher's exact test was used.

[#] Data available only in some cases.
